# Supplementary material for: Identification and functional analysis of non-coding regulatory small RNA FenSr3 in Bacillus amyloliquefaciens LPB-18
Source: PeerJ. 2023 May 15;11:e15236. doi: 10.7717/peerj.15236 (PMC10194069; doi:10.7717/peerj.15236)
Supplement: Supplemental Information 4 [file peerj-11-15236-s004.zip › KO/CK-vs-T1_map/map00908.html]

KEGG PATHWAY: Zeatin biosynthesis - Reference pathway


|  |  |
| --- | --- |
| **Zeatin biosynthesis - Reference pathway** |  |

[
Pathway menu
| Organism menu
| Pathway entry
| Show description
| User data mapping
]

Zeatin is a member of the cytokinin family, a class of phytohormones involved in various processes of growth and development in plants. Most abundant cytokinins are adenine-type, where the N6 position of adenine is substituted with an isoprenoid, such as in zeatin, or an aromatic side chain, such as in kinetin. Zeatin can be synthesized in two different pathways: the tRNA pathway and the AMP pathway. In the tRNA pathway zeatin is a recycled product of isopentenylated tRNAs. In the AMP pathway zeatin is synthesized from an isopentenyl donor, dimethylallyl diphosphate (DMAPP), and AMP, ADP, or ATP by isopentenyltransferases. After synthesis cytokinins can be glucosylated.

|  |  |  |
| --- | --- | --- |
| Reference pathway | 184% 150% 122% 100% 82% 67% 55% | 图片下载 |
